# Effectiveness of exercise interventions on androgen and sex hormone-binding globulin levels in women with polycystic ovary syndrome: a systematic review and meta-analysis

**DOI:** 10.3389/fspor.2025.1686566

**Published:** 2026-01-08

**Authors:** Leman Atmaca, Shalini Chauhan, Sachal Sadiq Najaf, Mohammed Elmadani, Maha Al-Jawarneh, Anaam Soloh, Zsuzsanna Varga, József Vitrai, Viktoria Prémusz

**Affiliations:** 1 Doctoral School of Health Sciences, Faculty of Health Sciences, University of Pécs, Pécs, Hungary; 2 GSY, Goodbye Stress with Yoga Project, University of Pécs, Pécs, Hungary; 3 Physical Education and Exercise Center, Medical School, University of Pécs, Pécs, Hungary; 4 Evidence-Based Medicine, Epistudia, Bern, Switzerland; 5 Department of Behavioral Sciences, Medical School, University of Pécs, Pécs, Hungary; 6 Department of Psychology and Healthcare Management, Faculty of Health and Sport Sciences, Széchenyi István University, Győr, Hungary; 7 Faculty of Health Sciences, Institute of Physiotherapy and Sports Science, University of Pécs, Pécs, Hungary; 8 National Laboratory on Human Reproduction, University of Pécs, Pécs, Hungary

**Keywords:** androgens, exercise, physical activity, polycystic ovary syndrome, sex hormone-binding globulin

## Abstract

**Introduction:**

Polycystic ovarian syndrome (PCOS) is a common disorder typified by hyperandrogenism, ovarian dysfunction, and metabolic disorders. Elevated androgen and decreased Sex Hormone Binding Globulin (SHBG) levels are some of the main features contributing to its clinical manifestations. Lifestyle interventions, especially exercise, are considered first-line management methods due to their positive effects on hormonal regulation. Due to a lack of comprehensively synthesized data on the effects of exercise on androgen and SHBG levels, in this systematic review and meta-analysis, we aimed to evaluate the effects of structured exercise on Total Testosterone and Dehydroepiandrosterone (DHEA-S) as primary parameters, and SHBG levels as secondary parameters.

**Methods:**

Relevant databases (EMBASE, Scopus PubMed, Web of Science, Cochrane Trials) were searched for Randomized Clinical Trials (RCTs) and Controlled Clinical Trials (CCTs) on exercise in Rotterdam-diagnosed PCOS women. Study selection and data extraction were carried out by two separate reviewers. Risk of bias was assessed using Cochrane RoB 2. Meta-analyses, employing a random-effects model, calculated Mean Differences (MDs) for hormonal outcomes, with subgroup analyses for different exercise types [e.g., aerobic, high-intensity interval training (HIIT)].

**Results:**

A total of 23 studies involving women with PCOS were included. For the overall effect of exercise vs. control, meta-analysis of 12 studies (*n* = 563 participants) showed no significant effect on Total Testosterone, DHEA-S (5 studies, *n* = 231 participants), or SHBG (10 studies, *n* = 422 participants). However, subgroup analysis revealed that aerobic exercise led to a statistically significant reduction in Total Testosterone levels (4 studies, *n* = 212 participants). HIIT did not show a significant effect on Total Testosterone or SHBG.

**Conclusion:**

Aerobic exercise significantly reduces total testosterone levels in women with PCOS, according to this meta-analysis, indicating that it may be used as a non-pharmacological method of managing hyperandrogenism.

**Systematic Review Registration:**

https://www.crd.york.ac.uk/PROSPERO/view/CRD42024502657, PROSPERO CRD42024502657.

## 1 Introduction

Polycystic ovarian syndrome (PCOS) is a prevalent endocrine condition that affects between 6% to 20% of women who are of reproductive age (1). It's characterized by hyperandrogenism, polycystic ovaries, and oligo or anovulation and is highly related to insulin resistance, obesity, and increased risk of cardiovascular and metabolic dysfunction (2). A hallmark feature of PCOS is acne, hirsutism, and alopecia caused by elevated androgen levels, mainly testosterone and androstenedione (3).

Sex hormone binding globulin (SHBG) has a crucial role in modulating the circulating free androgens. Often, SHBG levels in women with PCOS are decreased, which leads to an increase in hyperandrogenic symptoms and metabolic risks (4). The interplay between androgens and SHBG is complex; increased androgen production reduces hepatic SHBG synthesis, further amplifying free androgen levels and perpetuating hormonal imbalance (5).

Due to their effects on weight loss, insulin sensitivity, and positive effects on the various hormonal profiles, lifestyle interventions, especially exercise, are considered as first-line management strategies for PCOS (6). While exercise has been demonstrated to improve insulin resistance and decrease androgen levels, the extent and consistency of these effects have varied across individual studies. Some studies suggest that physical activity could also increase SHBG levels and potentially reduce the symptoms of hyperandrogenism (7). However, a comprehensive synthesis of the existing research to see these specific hormonal effects across different studies remains lacking.

Beyond the direct hormonal effects in PCOS, the broader field of sports and active living emphasizes the profound impact of regular physical activity on women's overall health and well-being, often highlighting its role in maintaining physiological balance and mitigating chronic disease risk. Understanding the specific mechanisms by which exercise influences intricate hormonal pathways, such as those involved in androgen and SHBG regulation, can not only inform targeted interventions for conditions like PCOS but also contribute to a more holistic approach to women's health throughout the lifespan (8, 9). Furthermore, exploring the diverse forms of exercise and their unique physiological demands may reveal nuanced effects on endocrine systems, paving the way for personalized exercise prescriptions that optimize hormonal outcomes and improve quality of life (10).

In order to fully understand how exercise therapies affect androgen and SHBG levels in women with PCOS, a systematic review and meta-analysis are necessary, given the variety of study designs and reported results in the existing literature. The specific goal of this review is to assess the associations between exercise and the levels of total testosterone, Dehydroepiandrosterone (DHEA-S), and SHBG in women with PCOS based on the most recent findings. This will help to clarify the hormonal advantages of exercise and guide clinical recommendations for PCOS management.

## 2 Methods

This systematic review was prospectively registered on the Prospero International Prospective Register of Systematic Reviews (CRD42024502657). It was conducted in accordance with current best-practice guidelines (11) and reported accordance with the Preferred Reporting Items for Systematic Reviews and Meta-Analyses (PRISMA) statement (12).

### 2.1 Eligibility criteria

Studies were selected based on the Population, Intervention, Comparator, Outcome, and Study Design (PICOS) criteria as shown in Table 1. For the population we included women in all ages, who were diagnosed with PCOS based on the Rotterdam criteria (13). To be able to maintain homogeneity in diagnostic approach, we excluded the studies that included women diagnosed by other criterias (e.g., NIH, AE-PCOS). Intervention included any type of structured exercise training, e.g., high-intensity interval training (HIIT), strength training, flexibility training, and continuous aerobic exercise. Comparator was defined as control or minimal intervention groups that received no therapy or received basic lifestyle advice. The primary outcomes of this study are changes in androgen levels, specifically Total Testosterone and DHEA-S, while the secondary outcome is changes in SHBG levels. We included only Randomized Clinical Trials (RCTs) and Controlled Clinical Trials (CCTs). We excluded animal studies, reviews, case reports, or studies that did not report the specified primary or secondary outcomes. Studies combining exercise with pharmacological interventions where the effect of exercise could not be isolated were also excluded.

**Table 1:** Inclusion and exclusion criteria, and search strategy.

Domain	Inclusion criteria	Exclusion criteria	Search strategy
Population	Female with confirmed diagnosis of PCOS (Rotterdam Criteria) who are not receiving drugs, without age restriction	– Female with uncertain or undiagnosed PCOS status. – Female who had undergone any surgical intervention related to PCOS – Pregnant or breastfeeding women	Polycystic Ovary Syndrome [MeSH PCOS [Title/Abstract] Oligomenorrhea Hyperandrogenism [MeSH] Stein Leventhal Syndrome [Title/Abstract]
Intervention	– Any structured physical activity program – Aerobic, Resistance, HIIT, Yoga. Duration > _8 weeks. – Supervised or home-based.	Pharmacological interventions without exercise – Alternative therapies without exercise – Interventions shorter than 8 weeks	Exercise therapy[mesh] OR Exercise[tiab] OR physical activity[tiab] OR physical exercise[tiab] OR isometric exercise[tiab] OR Aerobic Exercise[mesh] OR Resistance training[mesh] OR resistance exercise[tiab] OR strength training[tiab] OR Weight lifting strengthening programs[tiab] OR weight bearing exercise program[tiab] OR High intensity interval training[mesh] OR High intensity intermittent exercise[tiab] OR Muscle Stretching Exercise[mesh] OR sprint interval training[tiab] OR Endurance training[mesh] OR concurrent training[tiab] OR Functional training[tiab] OR Running[mesh] OR jogging[mesh] OR Swimming[mesh] OR Walking [mesh] OR Cycling[tiab] OR Yoga[mesh]
Comparison	– Studies with a non-exercising control group – Studies with baseline(pre-post) comparisons within the same group – Studies comparing different types of exercise interventions(e.g., HIIT vs. aerobic)	– Studies without any control or comparator – Studies comparing exercise with non-physical interventions only (e.g., diet, medication alone)	
Outcome	Testosterone, DHEA-S, Sex hormone-binding globulin, Luteinizing hormone (LH), Progesterone, AMH Follicle-stimulating hormone (FSH), Oestrogen, and Prolactin, insulin resistance, lipid profile, fasting blood glucose	Studies not reporting at least one of the specified outcomes or partially reporting outcome	
Study Design	Randomized clinical trials and controlled clinical trials	Cross-sectional, reviews, case reports, or purely observational studies	Randomized controlled trial [MeSH] Controlled clinical trial [MeSH] Clinical trial [MeSH]

### 2.2 Search strategy

A comprehensive systematic search was conducted across the following electronic databases: EMBASE, PubMed, Scopus, Web of Science, and Cochrane Central Register of Controlled Trials. The search strategy employed a combination of Medical Subject Headings (MeSH) terms and keywords related to “Polycystic Ovary Syndrome”, “PCOS”, “Exercise”, “Physical Activity”, “Androgens”, “Testosterone”, “DHEA-S”, and “SHBG”. The search was conducted up to 2024, and was restricted to English language, and human studies.

In addition to electronic database searches, we checked the reference lists of all included publications to identify any further relevant studies. Subject-matter experts were also contacted for additional information. Grey literature was searched, including Clinical Trials.gov.

### 2.3 Study selection

All the identified studies were submitted to Rayyan (Rayyan Systems Inc.) to remove duplicates and expedite the screening process. The study selection process was conducted in two phases by two independent reviewers (LA, SC). In the first phase, titles and abstracts were screened for relevance against the predefined eligibility criteria. Potentially relevant articles proceeded to the second phase, where full-text screening of the publications was done for final inclusion. Any disagreements between the two reviewers were resolved through discussion and consensus or by consultation with a third reviewer (ME). A PRISMA flow diagram is used to illustrate the study selection process.

### 2.4 Data extraction

A standardized, pre-piloted data extraction form was constructed to systematically gather relevant information from each included study. Data were extracted independently by two reviewers (LA, SC), with any discrepancies resolved through discussion or by a third reviewer. The extracted data included author, year of publication, country, study design, allocation method, and title as study characteristics. Participant characteristics were baseline age, body mass index (BMI), number of individuals in each group, and specific diagnostic criteria for PCOS (ensuring Rotterdam criteria were met). For intervention details, type of exercise (e.g., aerobic, resistance, combined), frequency, intensity, duration, and total program length were included. Comparator details were added as a description of the control group intervention. Outcome measures were extracted as mean and standard deviation (or data from which these could be calculated) of change from baseline or post-intervention values for Total Testosterone, DHEA-S, and SHBG for both intervention and control groups.

Data was managed using Microsoft Excel.

### 2.5 Risk of bias assessment

The methodological quality and risk of bias of the included Randomized Clinical Trials (RCTs) was evaluated using the most recent version of the Cochrane Risk of Bias tool for randomized trials (RoB 2). Two independent reviewers (LA, SC) performed the risk of bias assessment, and any discrepancies were resolved through discussion or by consultation with a third reviewer (VP).

### 2.6 Data synthesis and statistical analysis

For the systematic review, descriptive data concerning the quantity and characteristics of the included studies is presented. Where sufficient data were available, meta-analyses were performed for primary (Total Testosterone, DHEA-S) and secondary (SHBG) outcomes. Mean Differences (MDs) with 95% Confidence Intervals (CIs) were calculated for continuous outcomes, comparing exercise interventions against control groups.

Statistical heterogeneity between studies was assessed using the Chi-squared test and quantified using the *I*^2^ statistic. An *I*^2^ value of 0%–40% was considered low heterogeneity, 30%–60% moderate, 50%–90% substantial, and 75%–100% considerable heterogeneity (14). Because heterogeneity across exercise modalities was anticipated *a priori*, subgroup analyses based on exercise type (aerobic, HIIT, resistance) were pre-specified as the primary strategy to explore and explain between-study variability. This approach aligns with established recommendations for synthesizing complex intervention trials.

Separate meta-analyses were conducted as subgroup analyses to evaluate the effects of different exercise types on hormonal outcomes. Specifically, these included overall effects of exercise vs. control, aerobic exercise vs. control, and HIIT vs. control.

Publication bias was visually assessed using funnel plots for outcomes with sufficient studies.

All statistical analyses were performed using Review Manager (RevMan) software.

## 3 Results

### 3.1 Study selection and characteristics

**Figure 1:**
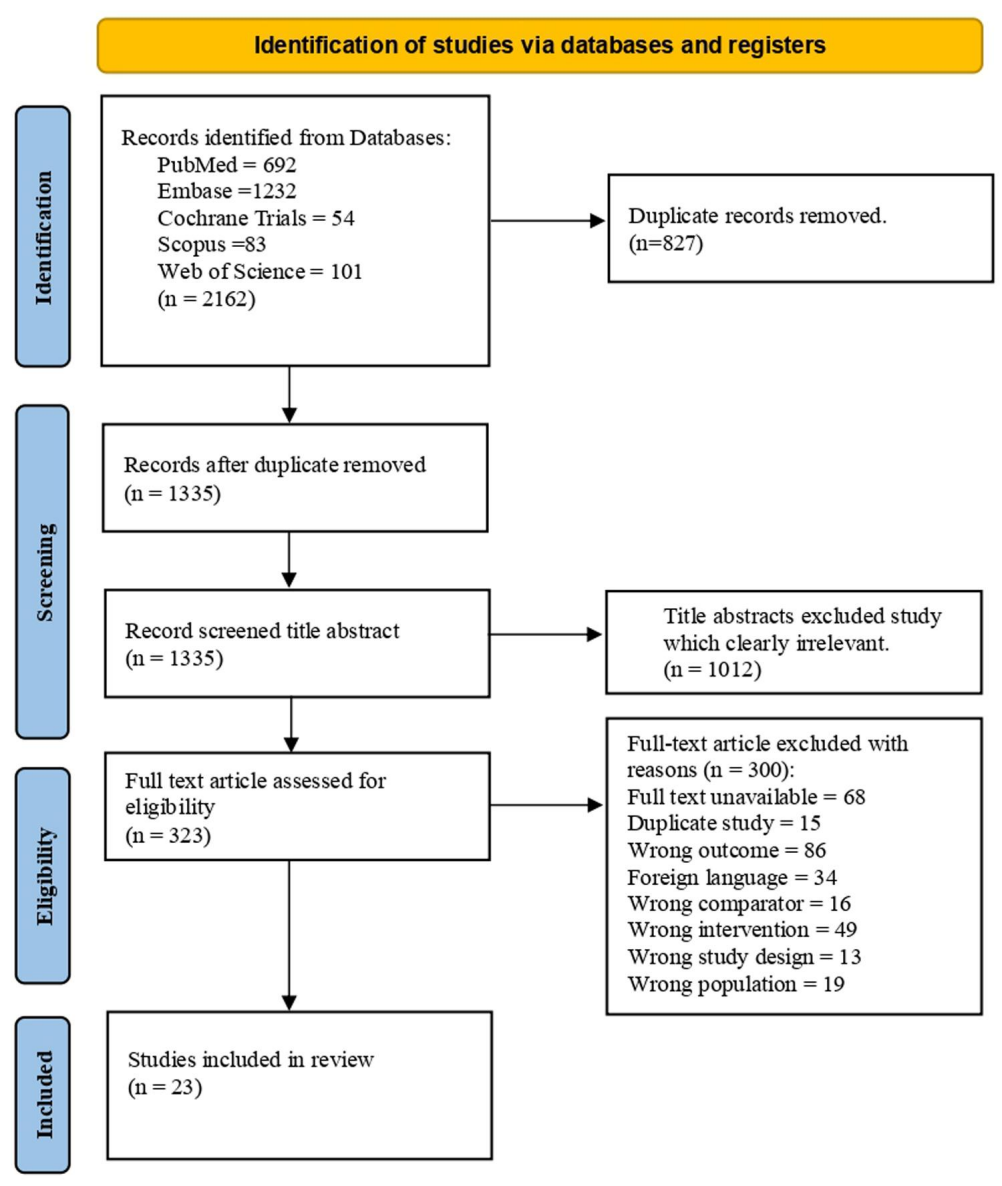
PRISMA flow diagram of study selection.

The systematic search across EMBASE, PubMed, Web of Science, Cochrane Central Register of Controlled Trials, Scopus, grey literature and list of references initially yielded 2,162 records. After removing 827 duplicate records, 1,335 unique titles and abstracts were screened. Of these, 1,012 records were excluded based on title and abstract, leaving 323 full-text articles for detailed assessment of eligibility. Following full-text review, 300 articles were excluded for various reasons (detailed in Figure 1). Ultimately, 23 studies met the predefined inclusion criteria and were included in the qualitative synthesis and meta-analysis. The study selection process is illustrated in the PRISMA flow diagram (Figure 1).

The included studies were conducted across various countries, ensuring a broad geographical representation. They all involved women diagnosed with Polycystic Ovary Syndrome exclusively based on the Rotterdam criteria, to ensure homogeneity of the study population. The sample sizes across the studies varied, and the exercise interventions encompassed diverse modalities including aerobic training, resistance training, HIIT, and combined approaches, with intervention durations ranging from 8 to 24 weeks/months. Key details such as participant demographics, specific intervention protocols (frequency, intensity, type), control group descriptions, and reported outcome measures are provided in detail in Supplementary Table S1.

### 3.2 Effect of exercise on androgen levels (total testosterone and DHEA-S)

**Figure 2:**
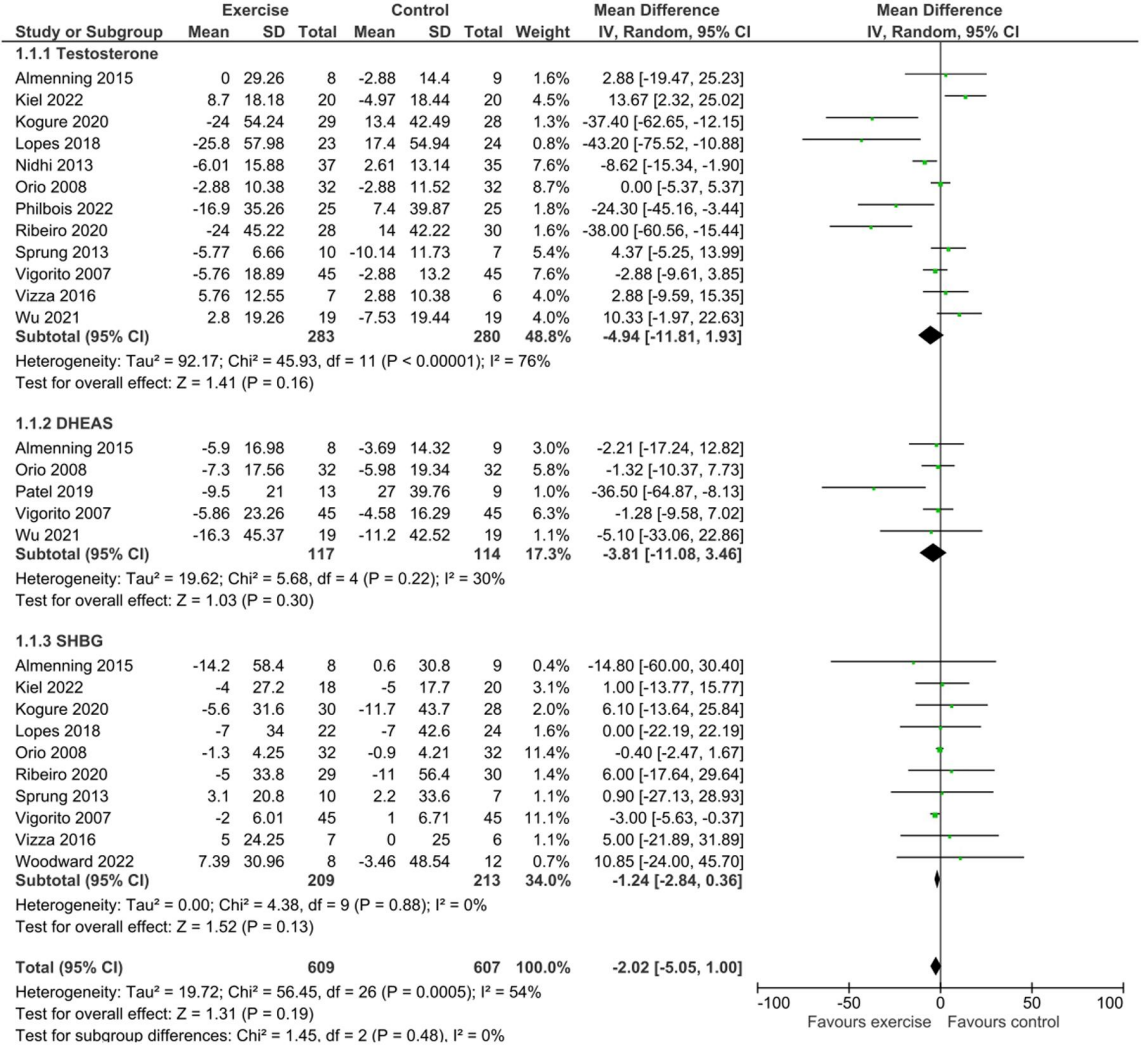
Forest plot of the overall mean difference in total testosterone, DHEA-S, and SHBG levels comparing any exercise intervention to control.^1^

The overall meta-analysis assessing the effect of any exercise intervention vs. control on Total Testosterone included 12 studies with 283 participants in exercise groups and 280 in control groups. The pooled mean difference for Total Testosterone was −4.94 (95% CI: −11.81, 1.93), indicating no statistically significant change (*Z* = 1.41, *P* = 0.16). Substantial heterogeneity was observed across studies (*I*^2^ = 76%, *P* < 0.00001). This overall effect is presented in Figure 2.

**Figure 3:**
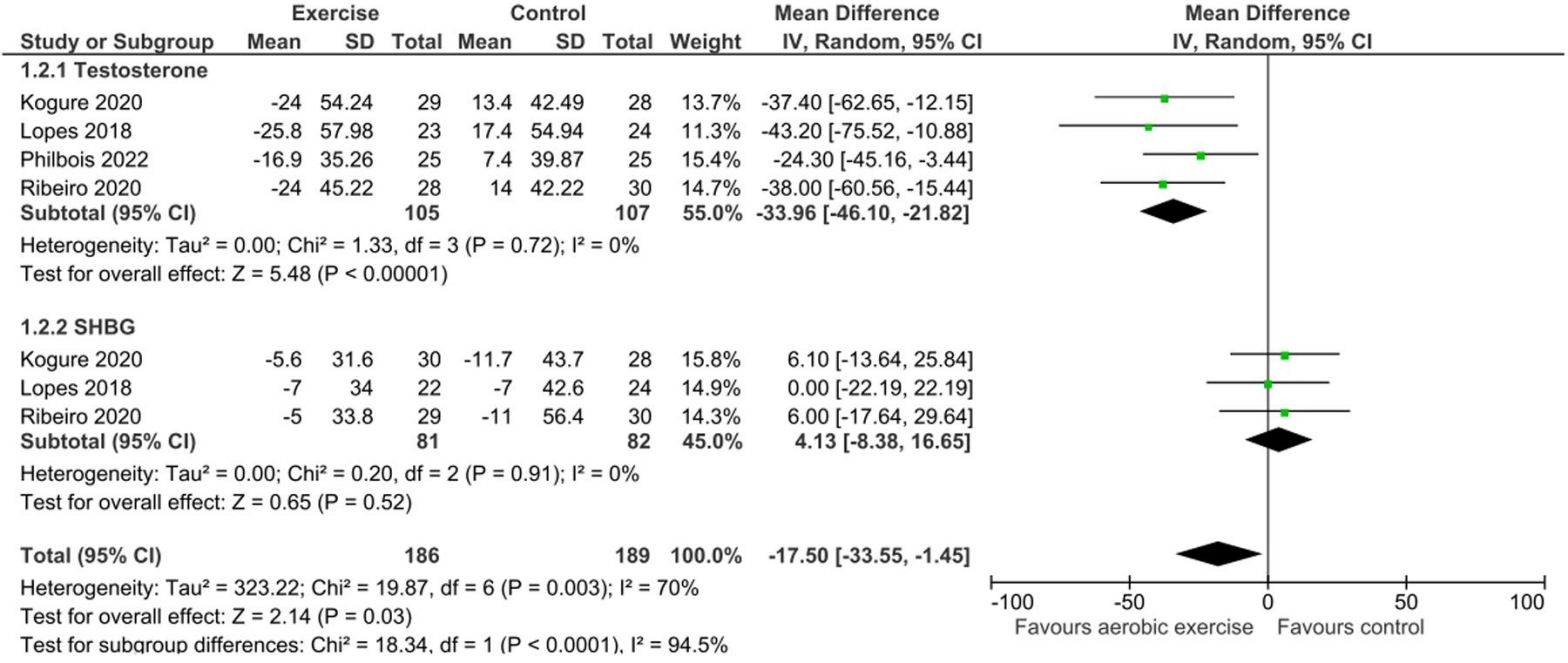
Forest plot of the mean difference in total testosterone and SHBG levels comparing aerobic exercise interventions to control^2^.

**Figure 4:**
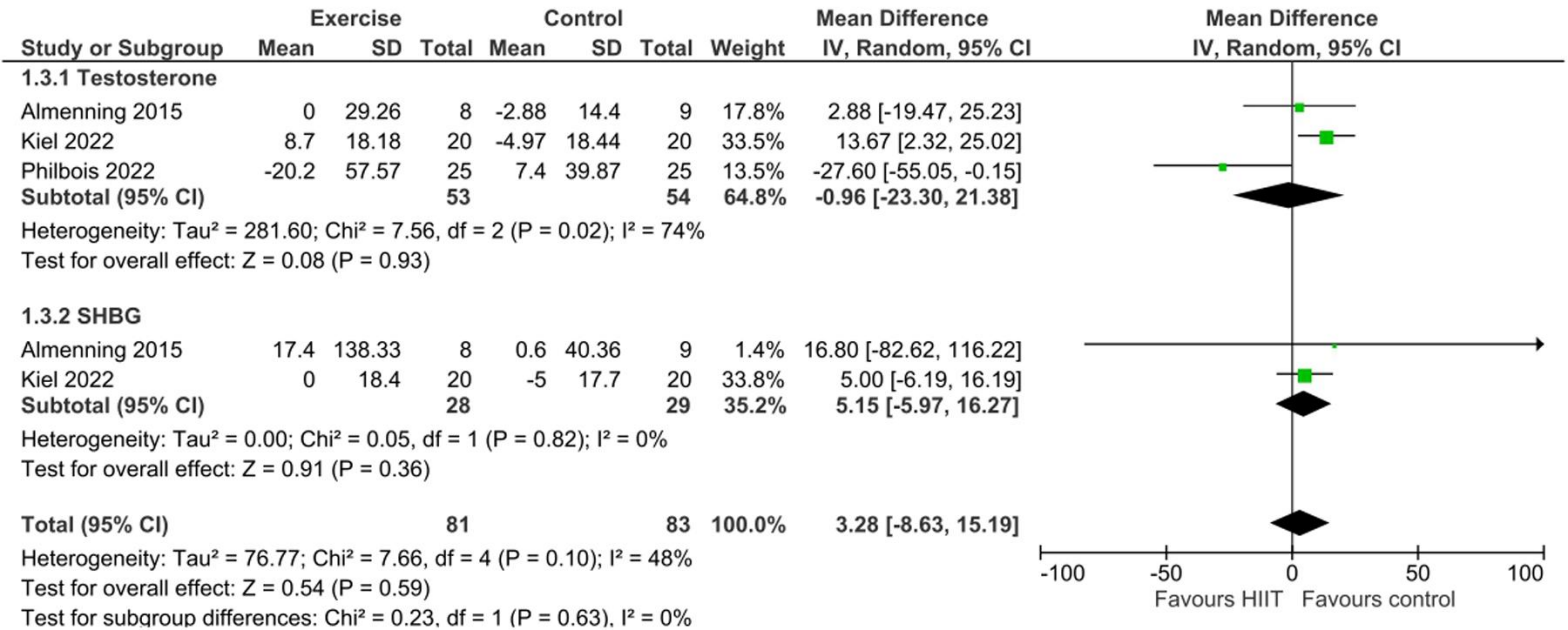
Forest plot of the mean difference in total testosterone and SHBG levels comparing HIIT to control^3^.

Subgroup analyses revealed varying effects based on exercise type for Total Testosterone. For aerobic exercise specifically, 4 studies involving 105 exercise participants and 107 control participants showed a statistically significant reduction in Total Testosterone levels (MD = −33.96, 95% CI: −46.10, −21.82; *Z* = 5.48, *P* < 0.00001). No significant heterogeneity was found within this subgroup (*I*^2^ = 0%, *P* = 0.72). This effect is illustrated in Figure 3. In contrast, HIIT, based on 3 studies (53 exercise participants, 54 control participants), did not result in a statistically significant change in Total Testosterone (MD = −0.96, 95% CI: −23.30, 21.38; *Z* = 0.08, *P* = 0.93). Substantial heterogeneity was observed for HIIT interventions (*I*^2^ = 74%, *P* = 0.02). This is shown in Figure 4.

For DHEA-S, the meta-analysis of 5 studies (117 exercise participants, 114 control participants) revealed no statistically significant overall effect of exercise (MD = −3.81, 95% CI: −11.08, 3.46; *Z* = 1.03, *P* = 0.30). Low heterogeneity was observed for DHEA-S (*I*^2^ = 30%, *P* = 0.22). The pooled effect for DHEA-S is included in Figure 2.

### 3.3 Effect of exercise on SHBG levels

The overall meta-analysis for SHBG included 10 studies with 209 exercise participants and 213 control participants. The pooled mean difference was −1.24 (95% CI: −2.84, 0.36), indicating no statistically significant change in SHBG levels following exercise interventions (*Z* = 1.52, *P* = 0.13). Importantly, no heterogeneity was observed for the overall effect on SHBG (*I*^2^ = 0%, *P* = 0.88). This result is part of Figure 2.

In subgroup analyses, aerobic exercise (3 studies, 81 exercise participants, 82 control participants) showed no statistically significant effect on SHBG levels (MD = 4.13, 95% CI: −8.38, 16.65; *Z* = 0.65, *P* = 0.52). No heterogeneity was detected (*I*^2^ = 0%, *P* = 0.91). This finding is presented in Figure 3. Similarly, HIIT, based on 2 studies (28 exercise participants, 29 control participants), did not significantly affect SHBG levels (MD = 5.15, 95% CI: −5.97, 16.27; *Z* = 0.91, *P* = 0.36), and no heterogeneity was observed (*I*^2^ = 0%, *P* = 0.82). This is shown in Figure 4.

### 3.4 Risk of bias within studies

**Figure 5:**
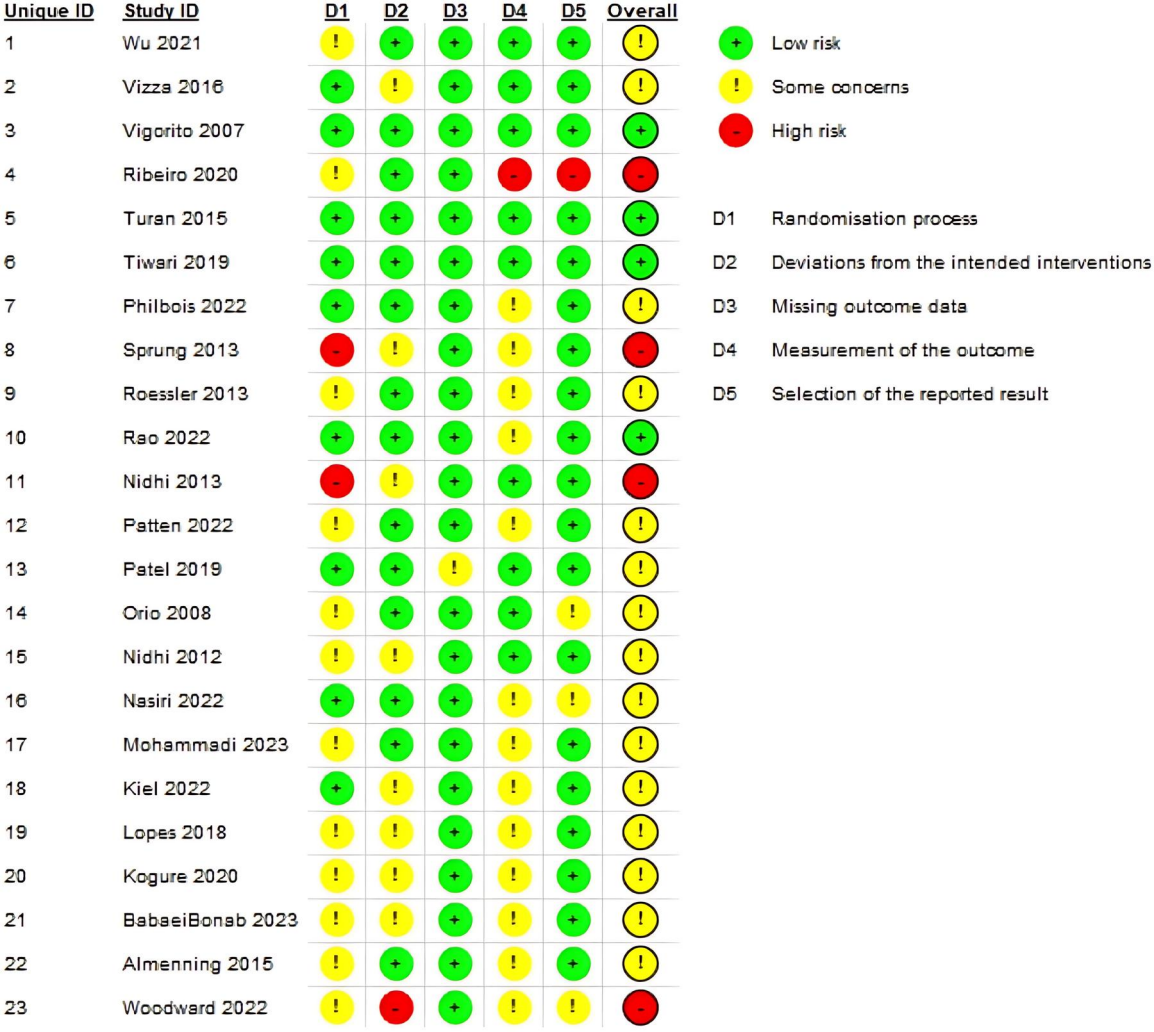
Detailed risk of bias judgments for individual studies included in the meta-analysis^4^.

**Figure 6:**
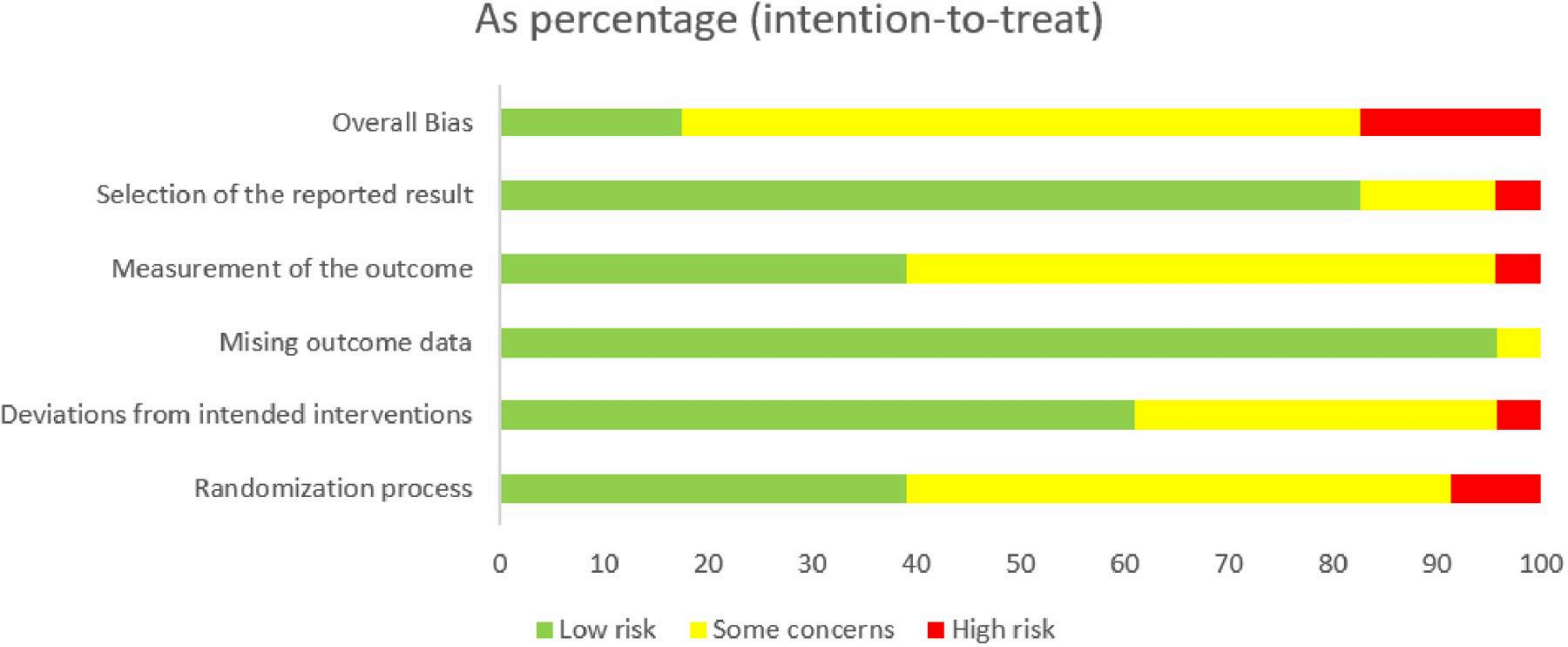
Aggregate risk of bias assessment for included studies, presented as percentages (intention-to-treat analysis)^5^.

The methodological quality and risk of bias for each included study were systematically assessed using the Cochrane Risk of Bias 2 (RoB 2) tool for randomized controlled trials. The aggregated results of the risk of bias assessment across all domains are summarized in Figure 5, and the detailed judgments for each study are presented in Figure 6.

Overall, the studies included exhibited varying levels of methodological quality. Most of the studies (82%) were categorized as having “some concerns” regarding their overall risk of bias. Importantly, upon detailed inspection of individual study judgments, four studies were identified as having an “overall high risk of bias” (e.g., Ribeiro 2020, Sprung 2013, Nidhi 2013, Woodward 2022), highlighting specific methodological limitations in these trials. The remaining 18% of studies were at “low risk of bias”.[Fn n3]

Upon closer examination of individual domains, the most prevalent sources of potential bias were observed in “Domain 1: Bias arising from the randomization process” and “Domain 2: Bias due to deviations from intended interventions”. Specifically, 64% of studies raised “some concerns” regarding the randomization process, often due to unclear reporting of allocation concealment or sequence generation. Similarly, 59% of studies had “some concerns” regarding deviations from intended interventions, largely due to the inherent difficulty in blinding participants and personnel in exercise interventions.

Conversely, studies generally performed well in other areas. A large majority of studies were at “low risk of bias” for “Domain 3: Bias due to missing outcome data” (82%), “Domain 4: Bias in measurement of the outcome” (68%), and particularly for “Domain 5: Bias in selection of the reported result” (91%). These findings suggest that while challenges related to the practical implementation and reporting of randomization and blinding in exercise trials exist, issues concerning data completeness and selective reporting were less common among the included studies.[Fn n4][Fn n5]

### 3.5 Sensitivity analysis

To assess the robustness of our findings, a sensitivity analysis was performed by re-running the meta-analyses after excluding studies identified as having an overall “high risk of bias” according to the Cochrane RoB 2 tool. The detailed results of these analyses are presented in Table 2.

**Table 2:** Sensitivity analysis Results.

Outcome	Analysis type	Number of studies (n)	Mean difference (95% CI)	Heterogeneity (*I*² %)	*P*-value
Testosterone (Overall)	Primary Analysis—All Studies	12	−4.94 (−11.81, 1.93)	76	—
	Sensitivity Analysis—Low/Some RoB Studies	9	−3.37 (−8.08, 1.34)	59	0.16
DHEA-S (Overall)	Primary Analysis—All Studies	5	−3.81 (−11.08, 3.46)	30	—
	Sensitivity Analysis—Low/Some RoB Studies	4	−3.10 (−10.53, 4.33)	35	0.42
SHBG (Overall)	Primary Analysis—All Studies	10	−1.24 (−2.84, 0.36)	0	—
	Sensitivity Analysis—Low/Some RoB Studies	7	−1.25 (−2.93, 0.44)	0	0.15
Testosterone (Aerobic)	Primary Analysis—All Studies	4	−33.96 (−46.10, −21.82)	0	<0.00001
	Sensitivity Analysis—Low/Some RoB Studies	4	−33.96 (−46.10, −21.82)	0	<0.00001
SHBG (Aerobic)	Primary Analysis—All Studies	3	4.13 (−8.38, 16.65)	0	0.52
	Sensitivity Analysis—Low/Some RoB Studies	3	4.13 (−8.38, 16.65)	0	0.52
Testosterone (HIIT)	Primary Analysis—All Studies	3	−0.96 (−23.30, 21.38)	74	—
	Sensitivity Analysis—Low/Some RoB Studies	2	8.41 (−1.72, 18.53)	17	0.10
SHBG (HIIT)	Primary Analysis—All Studies	2	5.15 (−5.97, 16.27)	0	0.36
	Sensitivity Analysis—Low/Some RoB Studies	2	5.15 (−5.97, 16.27)	0	0.36

For Total Testosterone, while the primary analysis showed no significant overall effect, its pooled mean difference slightly shifted towards zero (from −4.94 to −3.37) and heterogeneity notably decreased (from *I*^2^ = 76% to *I*^2^ = 59%) upon exclusion of high-risk-of-bias studies. Despite this, the findings remained non-significant. Similarly, the overall effects on DHEA-S and SHBG remained non-significant and largely robust to the exclusion of high-risk studies.

Crucially, the statistically significant reduction in Total Testosterone observed with aerobic exercise remained highly robust and unchanged after sensitivity analysis, as this subgroup did not include high-risk-of-bias studies. For HIIT, while the non-significant effect on Total Testosterone persisted after excluding a high-risk study, the mean difference shifted, and heterogeneity reduced (from *I*^2^ = 74% to *I*^2^ = 17%). Findings for SHBG in both aerobic and HIIT subgroups were consistently robust.[Fn n6]

### 3.6 Publication bias

**Figure 7:**
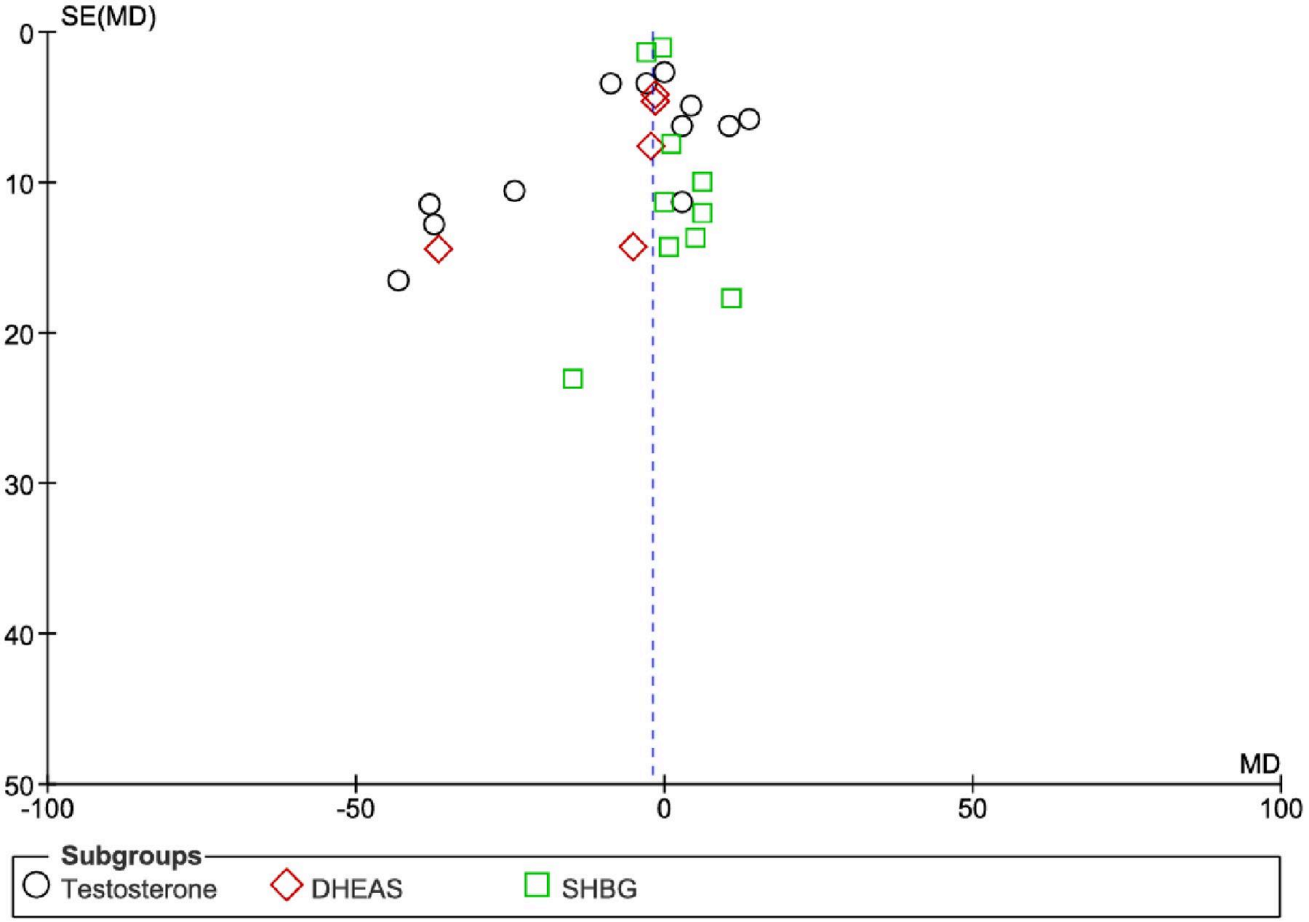
Funnel plot assessing publication bias for the overall effect of all exercise types on total testosterone (circles), DHEA-S (red diamonds), and SHBG (green squares)^6^.

A visual examination of the funnel plot was used to determine publication bias, which includes all exercise types for Total Testosterone, DHEA-S, and SHBG, as presented in Figure 7. Visually, the plot shows that most studies are clustered around and to the left of the zero line (Mean Difference = 0), indicating a tendency towards negative mean differences (reduction in hormone levels). While studies are generally distributed across various levels of precision (Standard Error of Mean Difference), there appears to be some asymmetry, with a noticeable absence of smaller studies (higher Standard Error of Mean Difference) showing positive or no effects on the right side of the plot. This observed asymmetry might suggest the presence of publication bias, where studies with non-significant or positive results are less likely to be published or identified.

## 4 Discussion

This systematic review[Fn n7] and meta-analysis focused on comprehensively evaluating the effect of structured exercise interventions on primary outcomes of Total Testosterone and DHEA-S levels, and the secondary outcome of SHBG levels, in women diagnosed with PCOS. Our findings partially support our hypothesis, indicating a nuanced effect of exercise on hormonal parameters in this population.

Our comprehensive meta-analysis aimed to elucidate the impact of various exercise interventions on hormonal parameters in women with PCOS. Initially, when considering all types of exercise interventions collectively, our findings indicated that exercise, in general, did not lead to a statistically significant change in Total Testosterone, DHEA-S, or SHBG levels when compared to control groups. It is therefore important to emphasize that a broad recommendation for “exercise” is not supported by the pooled hormonal outcomes, and the meaningful effect was isolated specifically within aerobic interventions. This distinction is now explicitly highlighted to avoid overgeneralizing the pooled findings. However, a more nuanced picture emerged from our subgroup analyses based on exercise modality. We observed a clear and statistically significant reduction in Total Testosterone levels specifically following aerobic exercise interventions.[Fn n8] This particular finding for aerobic exercise was notable for its consistency across studies, suggesting a robust and reliable effect. In contrast, interventions categorized as HIIT did not demonstrate a statistically significant impact on either Total Testosterone or SHBG in our pooled analysis. Given the substantial heterogeneity within HIIT studies and the limited number of available trials, these findings should be interpreted as inconclusive rather than reflective of a true absence of effect. Variation in HIIT protocol design (e.g., work–rest ratios, intensity definitions) likely contributed to this inconsistency.limi These results collectively underscore a crucial insight: while a broad recommendation for “exercise” may not uniformly affect hormonal markers in PCOS, specific modalities, such as aerobic exercise, appear to hold distinct therapeutic potential that warrants focused consideration in clinical practice.

When we compare our findings with previous literature, particularly the non-significant overall effect of exercise on Total Testosterone, DHEA-S, and SHBG, they align with some previous meta-analyses and systematic reviews that have also reported limited or inconsistent effects of general exercise interventions on these specific hormonal parameters in women with PCOS (15–17). However, the significant reduction in Total Testosterone observed specifically with aerobic exercise interventions provides a more nuanced understanding, suggesting that the type of exercise is a critical determinant of hormonal outcomes. This specific effect of aerobic exercise on hyperandrogenism is strongly supported by mechanistic insights suggesting improvements in insulin sensitivity, which is a key factor in the syndrome's pathophysiology and directly influences ovarian androgen production (18–20). High insulin levels stimulate the ovaries and adrenal glands to produce more androgens, and improving insulin sensitivity through aerobic exercise can mitigate this effect (21). While these pathways provide plausible biological explanations, mechanistic conclusions cannot be directly inferred from this meta-analysis and require dedicated experimental studies measuring ovarian and adrenal markers concurrently with hormonal and metabolic outcomes.

The absence of a significant effect of HIIT on Total Testosterone in our meta-analysis, despite its known efficacy in improving other metabolic parameters in PCOS like insulin resistance (22), contrasts with some smaller studies that might have suggested benefits. The small number of studies in our HIIT subgroup, the variation in HIIT protocols (e.g., intensity, duration, work-rest ratios), or the potential that the hormonal responses to HIIT are different from those to continuous aerobic activity in this cohort could all be reasons for this disparity.

Regarding SHBG, our meta-analysis indicates no significant change following any exercise intervention, which is consistent with several previous systematic reviews that have also reported a limited influence of exercise on SHBG levels in women with PCOS (16, 23). This suggests that while exercise can influence androgen production, its effect on SHBG, a key regulator of free androgen bioavailability, may be less direct or require different or more prolonged intervention strategies. The overall emphasis on lifestyle modifications, including physical activity, for the management of PCOS is well-established (24), resonating with broader public health efforts to promote healthy behaviors. The importance of understanding specific impacts of different physical activity types aligns with studies investigating diverse aspects of lifestyle and health, such as the role of physical activity in assisted reproductive therapy (25) or its connection to premenstrual syndrome and mental status (26). The varied responses to different exercise types further underscores the need for tailored interventions, a principle recognized in designing programs for other complex health challenges, such as multicomponent exercise for preventing falls in older adults (27). Discrepancies between reviews may also arise from differences in inclusion criteria (e.g., PCOS diagnostic criteria, study design), population characteristics, intervention duration, and methodological quality of included studies.

The observed hormonal effects of exercise in women with PCOS are likely mediated through a complex interplay of various biological mechanisms. The most prominent of these is the improvement in insulin sensitivity, a cornerstone of the syndrome's pathophysiology (28). Aerobic exercise, which our findings highlight for its significant effect on Total Testosterone, is known to be a potent intervention that enhances glucose uptake and increases insulin receptor sensitivity (29, 30). This reduces circulating insulin levels and thereby indirectly lessens the stimulatory effect of hyperinsulinemia on ovarian androgen production. While other mechanisms, such as changes in adiposity, inflammation, or SHBG synthesis, may also contribute to the observed hormonal improvements, these pathways require further direct mechanistic studies for complete clarification (31, 32).

When it comes to weight loss and body composition, while hormonal improvements can occur independent of significant weight loss, especially with exercise interventions (33), changes in body composition (specifically reduction in adiposity) can play a role in ameliorating hyperandrogenism in overweight and obese women with PCOS (34). Adipose tissue, particularly visceral fat, is metabolically active and can contribute to androgen synthesis and metabolism. It contains aromatase, an enzyme that converts androgens to estrogens (35). A reduction in adiposity can lead to lower aromatase activity, potentially decreasing the peripheral conversion of androgens to estrogens, thereby influencing circulating androgen levels (36). Furthermore, obesity in PCOS is associated with chronic low-grade inflammation, which can exacerbate insulin resistance and hyperandrogenism (37). A more balanced hormonal environment may be indirectly influenced by exercise-induced improvements in body composition and a decrease in inflammation (38).

Another mechanism we can talk about is direct gonadal and adrenal effects. While less studied as a primary mechanism compared to systemic metabolic changes, exercise may exert some direct influence on ovarian steroidogenesis. Improvements in blood flow, reduction in oxidative stress, and modulation of inflammatory markers within the ovaries could theoretically impact androgen production (39). However, direct evidence of exercise's independent effect on ovarian androgen production, separate from systemic metabolic changes, remains largely speculative and requires further investigation (40). Regarding adrenal androgens like DHEA-S, while DHEA-S levels are often elevated in women with PCOS, our meta-analysis found no significant overall effect of exercise on DHEA-S. This implies that exercise may have a less direct impact on adrenal androgen secretion compared to ovarian androgen production, or that the mechanisms affecting adrenal function are different and less responsive to general exercise interventions.

## 5 Conclusion

This systematic review and meta-analysis aimed to evaluate the effect of structured exercise interventions on androgen levels (Total Testosterone, DHEA-S) and SHBG in women with PCOS.

Our findings demonstrate that while overall exercise interventions did not significantly alter Total Testosterone, DHEA-S, or SHBG levels in women with PCOS, aerobic exercise specifically led to a statistically significant reduction in Total Testosterone levels. This suggests a specific benefit of aerobic exercise in managing hyperandrogenism in PCOS, which is a hallmark feature of the syndrome.

These results underscore the importance of distinguishing between different types of exercise when prescribing interventions for women with PCOS, particularly when targeting hormonal outcomes. The significant reduction in Total Testosterone with aerobic exercise highlights its potential as a targeted non-pharmacological strategy for improving endocrine profiles in this population.

In conclusion, our findings reinforce the role of aerobic exercise as a potent, non-pharmacological, and lifestyle-based intervention for managing hyperandrogenism in women with PCOS. The significant reduction in Total Testosterone levels observed with this modality provides a strong rationale for its inclusion in clinical guidelines. Future efforts should focus on encouraging the development of personalized exercise prescriptions based on individual clinical profiles and preferences. Moreover, to ensure sustained benefits, it is critical to improve long-term adherence. This can be achieved by integrating behavioral support strategies, structured follow-ups, and motivational interventions into physical activity programs. Further research should prioritize larger, well-designed randomized controlled trials that directly compare different exercise modalities with standardized protocols and longer follow-up periods to better understand their differential effects on hormonal parameters and other PCOS-related outcomes.

### 5.1 Clinical implications

The meta-analysis highlights the significant practical implications for PCOS management, particularly regarding exercise recommendations. Our key finding—that aerobic exercise specifically reduces Total Testosterone levels—suggests it should be a prioritized component in lifestyle interventions to manage hyperandrogenism (30). This refines general exercise advice, indicating a targeted approach to address hormonal imbalances. Exercise remains a cornerstone of non-pharmacological management, offering broad benefits beyond hormones, including improved insulin resistance and overall well-being (41, 42). Its role as a primary, accessible intervention is crucial, emphasizing the need for personalized exercise prescriptions to enhance adherence and achieve specific patient goals (43, 44). However, achieving sustained adherence remains a major challenge in this population due to factors such as body image concerns, motivational fluctuations, and fatigue. Integrating behavioral counseling, social support, and digital tools (e.g., activity tracking) may help improve long-term compliance with exercise regimens.

It should also be noted that the demonstrated hormonal benefit pertains specifically to reductions in Total Testosterone through aerobic exercise; effects on SHBG and DHEA-S were not significant, reinforcing the need for modality-specific recommendations in clinical practice.

### 5.2 Strengths and limitations

This meta-analysis benefits from a rigorous methodology, including a pre-registered PROSPERO protocol (CRD42024502657) and adherence to PRISMA guidelines, ensuring minimized bias. Our comprehensive search, focus on randomized controlled trials, and detailed risk of bias assessment enhance the reliability of our findings. The subgroup analyses were crucial for identifying the specific effect of aerobic exercise amidst the heterogeneity of interventions.

Despite these strengths, several limitations must be acknowledged. Inherent heterogeneity across exercise protocols exists, even within subgroups, and the relatively small number of studies for some outcomes, particularly DHEA-S and HIIT, may limit the statistical power of those specific analyses. Furthermore, a key limitation, common to the synthesized literature, is the inconsistent reporting of detailed patient-level clinical data across the included studies. While baseline age and morphometric status (BMI) were collected, crucial factors such as sports history, detailed reproductive health (e.g., infertility status), and type and duration of contraceptive use were often not reported, making it difficult to fully assess their potential influence on hormonal outcomes. Challenges common to exercise trials, such as blinding and potential for varying adherence, may also impact results (45). Our exclusion criteria, which omitted studies not reporting primary hormonal outcomes, mean our analysis does not fully capture the broader cardiometabolic benefits of exercise in PCOS (46). Finally, while funnel plots were examined, drawing definitive conclusions about publication bias was difficult due to limited study numbers.

### 5.3 Future research

Future research should prioritize larger, high-quality randomized controlled trials using standardized and clearly defined exercise protocols to evaluate hormonal outcomes in women with PCOS. Determining the optimal “dose” of aerobic exercise—specifically the frequency, intensity, and duration necessary to achieve meaningful hormonal improvements—remains a key clinical priority. Longitudinal studies are also needed to assess the sustainability of these effects beyond the intervention period.

To enhance the comparability and utility of future systematic reviews and meta-analyses, primary studies should standardize and comprehensively report detailed patient-level characteristics. These should include, in addition to age and anthropometric status, habitual physical activity or sports history, reproductive health variables (e.g., infertility status, parity, miscarriage history), and the type and duration of hormonal contraceptive or insulin-sensitizing medication use, given their direct impact on androgen and SHBG regulation.

Future studies would also benefit from concurrently measuring hormonal, metabolic, and inflammatory markers to clarify the mechanisms through which exercise exerts its effects. Investigating responses across specific PCOS phenotypes (e.g., lean vs. obese, insulin-resistant vs. insulin-sensitive) may help identify subgroups most likely to benefit from particular exercise modalities (47). Finally, well-designed comparative effectiveness trials directly contrasting aerobic, HIIT, and resistance training are needed to establish more precise, modality-specific exercise recommendations for clinical practice.

## Data Availability

Publicly available datasets were analyzed in this study. This data can be found here: The original contributions presented in the study are included in the article, further inquiries can be directed to the corresponding author.

## Footnotes

^1^The plot includes individual study results and the pooled effect for each hormone. The diamond represents the overall pooled mean difference and its 95% confidence interval for each outcome.

^2^The plot shows individual study results and the pooled effect for each outcome withing the aerobic exercise subgroup. The diamond represents the overall pooled mean difference and its 95% confidence interval.

^3^The plot displays individual study results and the pooled effect for each outcome within the HIIT subgroup. The diamond represents the overall pooled mean difference and its 95% confidence interval.

^4^Each study is assessed across five domains (D1–D5) using the Cochrane RoB 2 tool, with judgments categorized as low risk (green circle), some concerns (yellow exclamation mark), or high risk (red circle). The overall risk of bias for each study is also indicated.

^5^This bar chart summarizes the proportion of studies categorized as low risk, some concerns, or high risk of bias across each domain of the Cochrane RoB 2 tool, as well as the overall bias.

^6^The X-axis represents the Mean Difference (MD) in hormone levels, while the Y-axis represents the Standard Error of the Mean Difference (SE(MD)), with lower values indicating higher precision studies and the axis inverted for conventional funnel plot representation. Asymmetry in the plot, particularly an absence of smaller studies (higher SE(MD)) showing positive effects, may suggest the presence of publication bias.
