# Supplementary material for: Effectiveness of exercise interventions on androgen and sex hormone-binding globulin levels in women with polycystic ovary syndrome: a systematic review and meta-analysis
Source: Front Sports Act Living. 2026 Jan 8;7:1686566. doi: 10.3389/fspor.2025.1686566 (PMC12824002; doi:10.3389/fspor.2025.1686566)
Supplement: Supplementary file 1 [file Table1.docx]

Supplementary Material

**Supplementary Table 1: Characteristics of Included Studies**

| **Reference** | **Country** | **Blinding (Allocation method)** | **Sample Size (BMI)** | **Intervention Duration** | **Age group (PCOS diagnostic criteria)** | **Intervention** | **Outcome Measures** |
| --- | --- | --- | --- | --- | --- | --- | --- |
| Wu 2021 | China | Random allocation | Exercise group: 19/19 (23.8±3.0)  Control group: 19/19 (24.1±3.2) | 12 wks 4 sessions/ week 60 mins/ session | 18 - 40  (Rotterdam) | **Exercise Group:** 12-week exercise program, 4 times/week, 1 hour per session. Sessions included a 15-minute warm-up, 30-minute aerobic phase on a bicycle ergometer at VO2AT​, and a 15-minute cool-down. Supervised with continuous ECG monitoring.  **Control Group:** Maintained normal lifestyle. | BMI, AMH, FSH, LH, Testosterone, DHEA-S |
| Vizza 2016 | Australia | Random allocation | PRT:8/7 (41.3(12.5)) Control:7/6 (33.8(9.4)) | 12 wks 2 sessions/ week 60 mins/ session | 18-42 (Rotterdam) | **PRT (Progressive Resistance Training) Group:** Prescribed two supervised and two unsupervised (home-based) training sessions per week for 12 weeks. Supervised sessions lasted approximately 60 minutes and included a 5-minute warm-up and cool-down. PRT exercises included lat pull-down, leg curl, seated row, leg press, calf raise, chest press, split squat, shoulder press, biceps curl, triceps extension, and abdominal curl. Sets were performed to neuromuscular fatigue (8-12 repetitions maximum); loads were increased with strength gains. Two sets of each exercise were prescribed in the first 2 weeks, progressing to 3 sets from week 3 for most exercises. Home-based calisthenics were undertaken on non-PRT days, performing 3 sets x 10 repetitions each of exercises like lying external hip rotations, side leg raises, push-ups on knees, wall squats, oblique curls, and core stabilization exercises.  **Control Group:** Did not receive any PRT intervention and were instructed to continue with their current lifestyle and usual healthcare and medical treatments. | BMI, Weight, WC, Testosterone, Fasting Insulin, FBG, Hs-CRP, SHBG |
| Vigorito 2007 | Italy | Random allocation | Trained: 45/45 (29.3±2.9) Untrained: 45/45 (29.4±3.5) | 3 months 3 sessions/ week 30 mins/ session | Average age ~21-22 years (Rotterdam) | **PCOS-T (trained) group:** Underwent a 3-month structured exercise training (ET) program on a hospital ambulatory-based regimen. Training sessions were performed three times per week under continuous electrocardiographic monitoring, supervised by a cardiologist, physiotherapist, and graduate nurse. Each session included a 5-min warm-up, 30 min of exercise on a bicycle ergometer at 60-70% of maximal oxygen consumption (VO2max​), and a 5-min cool-down. Exercise workload was gradually increased.  **PCOS-UnT (untrained) group:** Did not undergo the structured ET program. They received general dietary and behavioral advice without a structured caloric restriction program, focusing on a healthy balanced meal plan (50% carbohydrates, 25% protein, 25% fat) and encouraging low glycemic index foods | BMI, WC, WHR, FSH, LH, Prolactin, Estradiol, Progesterone, Testosterone, DHEA-S, Fasting Insulin, FBG, HDL, LDL, TC, TG, SBP, DBP, Hs-CRP, SHBG |
| Ribeiro 2020 | Brazil | Random allocation | Cont Aerobic:28/28 (28.4 (5.6)) Int Aerobic:29/29 (28.7 (4.8)) Control:30/30 (29.1 (5.2)) | 16 wks 3 sessions/week  Progressive from 30 minutes in the first wk, to 50 minutes in the last wk | 18 - 39  (Rotterdam) | **CAT (Continuous Aerobic Training) Group:** 16-week treadmill exercise, 3 times/week, progressing from 30 to 50 min/session. Intensity maintained at 64%-77% of maximum heart rate (HRmax).  **IAT (Intermittent Aerobic Training) Group:** 16-week treadmill exercise, 3 times/week, progressing from 30 to 50 min/session. Involved alternating periods of higher and lower intensity, ranging from 50% to 94% HRmax.  **Control Group (CG):** Maintained their usual daily physical activity. All participants in all groups were instructed to maintain their daily diets. | BMI, Weight, WC, HC, WHR, FSH, LH, Estradiol, Testosterone, FI, FBG, HDL, LDL, TC, TG, SHBG |
| Turan 2015 | Turkey | Random allocation | Training:14/14 (21.8±1.0)  Control:16/16 (21.9±1.1) | 8 wks 3 sessions/week  60 mins/ session | 17 – 34  (Rotterdam) | **Training Group:** 8-week structured exercise, 3 times/week. Each 50-60 minute session included warm-up, aerobic (step training, progressing to 20 min, 65-70% max HR) and resistance exercises (elastic bands, 15 reps, "somewhat intense"). Supervised by a physiotherapist.  **Control Group:** Did not participate in a structured exercise program.  All participants received general dietary advice. | BMI, WC, HC, FSH, LH, Estradiol, Testosterone, FI, FBG, HDL, LDL, TC, TG, SBP, DBP |
| Tiwari 2019 | India | Block Randomization  Double-blinded | Exercise w OP:33/33 (26.32±3.68)  Exercise w Met:33/33 (25.23±4.64) | 24 wks  3 sessions/week 30 mins/ session | 15-49 (Rotterdam) | **Exercise with Oral Placebo Group:** Followed a fixed exercise schedule (moderate intensity, 30 minutes, 3 days/week) along with an oral placebo (identical looking tablets).  **Exercise with Oral Metformin Group:** Followed the same fixed exercise schedule (moderate intensity, 30 minutes, 3 days/week) along with oral metformin (500mg, thrice daily with meals).  Both groups also received lifestyle advice regarding diet and exercise. | BMI, WC, WHR, Testosterone, TC, TG |
| Philbois 2022 | Brazil | Random allocation | MICT:25/25 (27.7±5.7)  HIIT:25/25 (27.8±4.2)  Control:25/25 (29.2±5.4) | 16 wks 3 sessions/week 60 mins/ session | 18 – 39 (Rotterdam) | **MICT (Moderate-Intensity Continuous Training) Group:** 16-week supervised continuous aerobic treadmill exercise, 3 times/week. Sessions (60 mins) included warm-up/cool-down, with 30-50 minutes at 64-77% of maximum heart rate (HRmax).  **HIIT (High-Intensity Interval Training) Group:** 16-week supervised interval aerobic treadmill exercise, 3 times/week. Sessions (60 mins) included warm-up/cool-down, with 30-50 minutes of alternating high (80-94% HRmax) and low (50-60% HRmax) intensity.  **Control Group:** Maintained their habitual level of physical activity. All participants maintained their daily diets. | BMI, Weight, Testosterone, FI, FBG, HDL, LDL, TC, TG, SBP, DBP, |
| Sprung 2013 | United Kingdom | Patient choice | Exercise:10/10 (31 (28 to 34))  Control:7/7 (35 (31 to 40)) | 16 weeks (Weeks 1-12: 3 sessions/week, 30 min/session; Weeks 13-16: 3 sessions/week, 45 min/session) | 18>  (Rotterdam) | **Exercise Group:** 16-week supervised moderate-intensity (~60% peak VO2​) exercise on a cycle ergometer or treadmill, 3 times/week. Session duration increased from 30 to 45 minutes after 12 weeks.  **Control Group:** Maintained habitual physical activity. | BMI, Weight, WC, FSH, LH, Estradiol, Progesterone, Testosterone, FI, FBG, HDL, LDL, TC, TG, SHBG |
| Roessler 2013 | Denmark | Random allocation | AE-GC:9/8 (36.7 (2.8)) GC-AE:9/8 (36.0 (2.3)) | 16 weeks (divided into two 8-week periods with a 4-week washout) 3 sessions/week | 19 – 46 (Rotterdam) | **AE-GC Group:** 8 weeks of Aerobic Exercise (AE) followed by 8 weeks of Group Counselling (GC). AE: 3 supervised sessions/week (5 min warm-up, 35-45 min cycle/treadmill at 60–85% VO2max​, 5 min cool-down). GC: weekly sessions on diet (Nordic Nutrition Recommendations) and increased daily physical activity.  **GC-AE Group:** 8 weeks of Group Counselling (GC) followed by 8 weeks of Aerobic Exercise (AE).  A 4-week washout period separated the two 8-week intervention periods. | BMI, Weight, WC |
| Rao 2022 | Pakistan | Random allocation | HIIT:25/20 (25.3±1.96) Strength:25/20 (26.5±3.09) | 12 wks  3 sessions/week | 20-40 (Rotterdam) | **HIIT (High-Intensity Interval Training) Group:** Performed HIIT 3 times/week for 12 weeks. Protocol included 4-minute high-intensity intervals (85-95% HRmax) interspersed with 3 minutes of active recovery (60-70% HRmax), repeated 4 times, with warm-up and cool-down.  **Strength Training Group:** Performed strength training 3 times/week for 12 weeks. Included exercises for major muscle groups, 3 sets of 8-12 repetitions at 70-80% of one-repetition maximum (1RM). | BMI, Testosterone |
| Nidhi 2013 | India | Random allocation | Yoga:45/37 (20.36(2.06)) Control:45/35 (21.10(2.98)) | 12 wks 1 hour/day | 15 – 19 (Rotterdam) | **Yoga Group:** Practiced a holistic yoga module (including asanas, pranayama, dhyana, yogic kriyas) for 1 hour/day, 12 weeks. Supervised sessions.  **Control Group:** Practiced conventional physical exercises (warm-up, stretching, aerobic exercises like spot jogging/skipping, free-hand exercises) for 1 hour/day, 12 weeks. | BI, Weight, AMH, FSH, LH, Prolactin, Testosterone |
| Patten 2022 | Australia | Random allocation | HIIT:15/13 (35.5±6.8)  MICT:14/11 (38.4±9.3) | 12 wks 3 sessions/week | 18 – 45  (Rotterdam) | **HIIT (High-Intensity Interval Training) Group:** 12-week supervised cycling, 3 times/week. Sessions included 5 min warm-up/cool-down, and 4 × 4 min intervals at 90–95% peak HR with 3 min active recovery (60–70% peak HR).  **MICT (Moderate-Intensity Continuous Training) Group:** 12-week supervised cycling, 3 times/week. Sessions included 5 min warm-up/cool-down, and 40 min continuous cycling at 60–70% peak HR.  Both groups received basic dietary advice. | BMI, Weight, WC, HC, WHR, AMH, Estradiol, Testosterone, FI, FBG, SHBG |
| Patel 2019 | USA | Random Allocation | Yoga: 16/16 (35.1 (1.5))  Control: 15/15 (35.4 (3.3)) | 12 wks  3 sessions/week 60 mins/ session | 23-42  (Rotterdam) | **Intervention Group (Mindful Yoga):** Practiced mindful yoga for 60 minutes, 3 times/week for 8 weeks. Sessions included gentle movements, breathwork, and meditation.  **Control Group:** Received no intervention, maintaining their usual lifestyle. | BMI, WHR, Testosterone, DHEA-S, FI, FBG |
| Orio 2008 | Italy | Random allocation | Trained:32/32 (28·9 ± 3·0) De-trained:32/32 (28·9 ± 2·3) | 24 wks 3 sessions/week 30ish minutes/session | Mean age 21.7 ± 2.3 years (Rotterdam) | **PCOS-T (trained) group:** Followed a 24-week supervised exercise training program (3 sessions/week, 30 min aerobic exercise at 60-70% of VO2max​ on a bicycle ergometer). This study then evaluated the effects of a subsequent 12-week detraining period (cessation of structured exercise).  **PCOS-UnT (untrained) group:** Acted as a comparison group, maintaining their usual lifestyle without structured exercise. | BMI, WC, WHR, FSH, LH, Prolactin, Estradiol, Progesterone, Testosterone, DHEA-S, Fasting Insulin, FBG, HDL, LDL, TC, TG, SHBG |
| Nidhi 2012 | India | Random allocation | Yoga:42/35 (20.30±1.92) Conventional PE:43/36 (21.22±2.99) | 12 wks 1 hour/day | 15 – 18 (Rotterdam) | **Yoga Group:** Practiced a yoga program (Suryanamaskara, asanas, pranayama, meditation) for 1 hour/day, every day for 12 weeks.  **Conventional Physical Exercise Group:** Practiced conventional physical exercises for 1 hour/day, every day for 12 weeks. | BMI, WC, HC, WHR, FI, FBG, HDL, LDL, TC, TG |
| Nasiri 2022 | Iran | Random allocation | Resistance:15/15 (29.9 ± 4.3) HIIT:15/15 (29.3 ± 4.3) Control:15/15 (30.7 ± 3.7) | 8 wks 3 sessions/week | 18 – 40 (Rotterdam) | **HIIT (High-Intensity Interval Training) Group:** 8-week exercise, 3 sessions/week. Each session: 5 min warm-up, 4 × 4 min intervals at 90-95% HRmax with 3 min active rest at 50-60% HRmax, 5 min cool-down.  **COM (Combined Training) Group:** 8-week exercise, 3 sessions/week. Each session: 30 min resistance training (3 sets of 8-12 reps at 70-80% 1RM for major muscles) followed by 30 min continuous aerobic exercise (60-70% HRmax).  **Control Group:** Maintained usual lifestyle. | BMI, Weight, WHR |
| Mohammadi 2023 | Iran | Random allocation | HIIT:14/14 (29.5 ± 4.5) Control:14/14 (31.4 ± 2.6) | 8 wks 3 sessions/week | 18 – 40 (Rotterdam) | **HIIT (High-Intensity Interval Training) Group:** 8-week supervised training, 3 sessions/week. Protocol involved 100-110% of maximum aerobic velocity (MAV), with 4-6 sets and 4 laps.  **Control Group:** Maintained normal daily activities without structured exercise. | BMI, Weight, WHR, FI, FBG, HDL, LDL, TC, TG |
| Kiel 2022 | Norway, Australia, United Kingdom | Random allocation | high-volume HIT :20/20 (85.4±22.6) low-volume HIT :21/18 (84.0±16.8) Control:23/20 (86.0±20.2) | 16 wks 3 sessions/week | 18-45 (Rotterdam) | **High-volume HIT Group:** 16-week supervised treadmill running, 3 sessions/week. Sessions included 10 min warm-up, 4 × 4 min intervals at 85–95% peak HR with 3 min active recovery at 70% peak HR, and 5 min cool-down.  **Low-volume HIT Group:** 16-week supervised treadmill running, 3 sessions/week. Sessions included 10 min warm-up, 1 × 4 min interval at 85–95% peak HR, and 5 min cool-down.  **Control Group:** Maintained habitual physical activity. | BMI, WC, HC, WHR, AMH, Progesterone, Testosterone, Fasting Insulin, HDL, LDL, TC, TG, SBP, DBP, SHBG |
| Lopes 2018 | Brazil | Random allocation | Cont Aerobic:23/23 (29.3(5.6)) Int Aerobic:22/22 (29.0(4.8)) Control:24/24 (29.9(5.3)) | 16 wks 3 sessions/week | 18-39 (Rotterdam) | **Continuous Aerobic Training (CAT) Group:** 16-week supervised exercise, 3 sessions/week. Sessions included 5 min warm-up, 40 min continuous aerobic exercise on a treadmill at 60-70% HRmax, and 5 min cool-down.  **Intermittent Aerobic Training (IAT) Group:** 16-week supervised exercise, 3 sessions/week. Sessions included 5 min warm-up, 4 × 4 min intervals at 85-95% HRmax with 3 min active recovery at 60-70% HRmax, and 5 min cool-down.  **Control Group:** Maintained normal lifestyle. | BMI, Weight, WHR, Testosterone, SHBG |
| Kogure 2020 | Brazil | Random allocation | Control:37/28 (29.1(5.2)) CAT:35/29 (28.4(5.6)) IAT:38/30 (28.6(4.7)) | 16 weeks 3 sessions/week | 18-39 (Rotterdam) | **Continuous Aerobic Training (CAT) Group:** 16-week supervised exercise, 3 sessions/week. Each session: 5 min warm-up, 40 min continuous aerobic exercise on a treadmill at 60-70% HRmax, and 5 min cool-down.  **Intermittent Aerobic Training (IAT) Group:** 16-week supervised exercise, 3 sessions/week. Each session: 5 min warm-up, 4 × 4 min intervals at 85-95% HRmax with 3 min active recovery at 60-70% HRmax, and 5 min cool-down.  **Control Group:** Maintained normal lifestyle. | BMI, Weight, WC, HC, WHR, Testosterone, SHBG |
| BabaeiBonab 2023 | Iran | Random allocation | Exercise:23/20 (28.16 ± 0.8) Control:23/20 (28.57 ± 0.07) | 12 wks  3 sessions/week | 14 - 18  (Rotterdam) | **Exercise Group:** Performed aerobic exercise for 12 weeks, 3 sessions/week. Each 60-minute session included 10 min warm-up, 40 min aerobic exercise (walking/running at 60-75% HRmax), and 10 min cool-down.  **Control Group:** Maintained usual daily activities. | BMI, Weight, HC, Prolactin, Testosterone, HDL, LDL, TC, TG, |
| Almenning 2015 | Norway | Computer Generated | Strength:11/8 (27.4±6.9) HIIT:10/8 (26.1±6.5) Control:10/9 (26.5±5.0) | 10 wks 3 sessions/week | NM (Rotterdam) | **HIIT (High Intensity Interval Training) Group:** 10-week supervised training, 3 sessions/week. Each session: 10 min warm-up, 4 × 4 min intervals at 85-95% maximal heart rate with 3 min active recovery at 70% maximal heart rate, 5 min cool-down (treadmill walking/running or cycling).  **ST (Strength Training) Group:** 10-week supervised training, 3 sessions/week. Each session: 10 min warm-up, 10 resistance exercises (3 sets of 10 repetitions with increasing load), 5 min cool-down.  **Control Group:** Maintained habitual physical activity. | BMI, Weight, WC, AMH, Testosterone, DHEA-S, Fasting Insulin, FBG, HDL, LDL, TG, SHBG |
| Woodward 2022 | United Kingdom | Random allocation | Sup exercise:12/8 (35.8(8.0)) Lifestyle PA:12/11 (35.1(8.5)) Control:12/12 (32.1(7.3)) | 12 wks 3 sessions/ week 60 mins/ session | 18> (Rotterdam) | **Exercise Group (EG):** 12-week supervised aerobic exercise training (two supervised, two unsupervised sessions per week). Supervised sessions involved 35 minutes of continuous moderate-intensity aerobic exercise (60-75% heart rate reserve) on treadmills, cycles, or cross-trainers, plus warm-up and cool-down. Unsupervised sessions were 30 minutes of moderate-intensity aerobic exercise. Heart rate monitors were used for real-time feedback.  **Lifestyle Physical Activity (LPA) Group:** 12-week intervention aiming to increase daily physical activity, using individual counseling based on Self-Determination Theory. Physical activity was monitored with accelerometers, with participants encouraged to meet UK physical activity guidelines (e.g., 150 minutes of moderate-intensity activity per week). | BMI, WC, HC, WHR, Fasting Insuin, FBG, HDL, LDL, TC, SHBG, |
